# A cross-sectional study on the pulmonary function of residents in two urban areas with different PM_10_ concentrations: data from the fourth Korea national health and nutrition examination survey (KNHANES) 2007–2009

**DOI:** 10.1186/s40557-018-0258-4

**Published:** 2018-07-16

**Authors:** Si Woo Park, Byoung Gwon Kim, Jung Woo Kim, Jung Woo Park, Jung Il Kim

**Affiliations:** Department of Occupational & Environmental Medicine, College of Medicine, Dong-A University, Busan, South Korea

**Keywords:** Airborne particulate matter, Pulmonary function test, Cross-sectional study, KNHANES

## Abstract

**Background:**

The present study aims to compare the pulmonary function of residents of Seoul special city (Seoul) and Jeju special self-governing province including Jeju city and Seogwipo city (Jeju), characterized by vastly different annual average airborne particulate matter with an aerodynamic diameter less ≤10 μm (PM_10_) concentrations, with the annual average PM_10_ concentration in Seoul being significantly higher than that in Jeju.

**Methods:**

This cross-sectional study analyzed the pulmonary function test results and sociodemographic data of Korean adults ≥19 years of age derived from the 4th KNHANES, 2007–2009. A total of 830 individuals residing in Seoul or Jeju were included in this study. T-tests were used to analyze predicted values of forced expiratory volume in 1 sec (FEV1p), predicted values of forced vital capacity (FVCp) and FEV1/FVC ratio (FEV1/FVC), as dependent variables, to examine the differences in the subjects’ pulmonary function according to the city of residence. Stratified analysis was then performed to adjust for variables potentially affecting pulmonary function. The analysis was performed on subjects as a group and also following stratification according to sex and other variables.

**Results:**

Seoul residents had a significantly lower FVCp than that of the Jeju residents (difference: 3.48%, *p* = 0.002). FEV1p, FVCp and FEV1/FVC of male Seoul residents were significantly lower than those of male Jeju residents (difference: 6.99, 5.11% and 0.03, respectively; *p* < 0.001, *p* = 0.001, *p* = 0.001). In male subjects, statistically significant results were obtained even after adjusting the influence of other variables through stratified analysis.

**Conclusion:**

The present analysis was based on cross-sectional data collected at one point in time. Therefore, unlike longitudinal studies, it does not establish a clear causal association between the variables. Nevertheless, this study found that pulmonary function among subjects residing in Seoul was significantly decreased compared to that of subjects residing in Jeju.

## Background

Airborne particulate matter, which includes dust, dirt, soot, smoke, and liquid droplets emitted into the air, is small enough to be suspended in the atmosphere. This complex mixture includes both organic and inorganic particles [1]. These particles vary greatly in size. PM_10_ includes both the coarse particle (size between 2.5 and 10 μm) and fine particles (measuring less than 2.5 μm) [2]. Most routine air quality monitoring systems generate data based on the measurement of PM_10_ as opposed to other airborne particulate matter sizes [3].

The Great Smog of London in 1952 was a severe air pollution event which resulted in approximately 4000 deaths [4] and drew the public’s attention to air pollution as a serious health hazard. Following this event, a series of epidemiological studies concerning the effects of air pollution on human health were conducted. A study by Samet et al. [5] investigated the link between mortality and air pollutants, including PM_10_, in 20 U.S. cities between 1987 and 1994. The study found that PM_10_ correlated with overall mortality, but also with mortality due to respiratory diseases, even after adjusting for other pollutants.

Recent trends of increased mortality from respiratory disease are due to acute exacerbation of pre-existing respiratory conditions triggered by PM_10_. According to a 2015 meta-analysis published by the Korea Centers for Disease Control and Prevention (KCDC) [6], an 10 μg/m^3^ increase in PM_10_ concentration increased hospitalization rates of patients with chronic obstructive pulmonary disease (COPD) by 2.7% (95% confidence interval [CI], 1.9–3.6%) and mortality by 1.1% (95% CI, 0.8–1.4%). In 1995, Norris et al. [7] investigated emergency room attendances for asthma among children over a 15-month period and found a strong correlation between the attendance rate and PM_10_ concentration (relative risk, 1.15; 95% CI, 1.08–1.23).

The chronic health hazards due to PM_10_ are less well understood than their acute health hazards. A number of studies have examined the steady reduction in pulmonary function and the increase in COPD occurrence. The Swiss Study on Air Pollution and Lung Disease in Adults (SAPALDIA) [8], followed-up 9651 adults aged 18–60 years across 8 Swiss regions for 11 years and found a significant negative correlation between decreases in the average annual PM_10_ concentration and FEV1 and FEV1/FVC. In the German study on the Influence of Air Pollution on Lung Function, Inflammation, and Aging (SALLIA) [9] followed-up 4757 women residing in Germany between 1985 and 1994 and found that a 7 μg/m^3^ increase in PM_10_ concentration over a five-year period was associated with a 5.1% reduction in FEV1 (95% CI, 2.5–7.7%), a 3.7% reduction in FVC (95% CI, 1.8–5.5%), and an increased odds ratio of 1.33 (95% CI, 1.03–1.72), suggesting that prolonged exposure to elevated PM_10_ concentration may have to do with development of COPD. However, a recent meta-analysis [10] of adult patients with COPD and PM_10_ concentration only found a statistically significant correlation among women and further research is required to investigate this association.

A number of Korean studies have examined the health hazards of PM_10_. However, most of the studies have focused on acute health hazards, such as asthma or exacerbations of COPD symptoms, with limited research on the chronic health effects. Furthermore, there are scarce studies based on regional comparisons. This study analyzed the data obtained from the Annual Report of Ambient Air Quality in Korea and the forth KNHANES to examine pulmonary function in Korean adults according to the average annual PM_10_ of the communities in which they reside.

## Methods

### Study subjects

The KCDC introduced the KNHANES in 1998. KNHANES aims to evaluate the nation’s health and nutritional status using a nationally representative sample. The 4th survey was conducted between 2007 and 2009 and the 2005 census data was used to determine a sample, stratified according to geography, sex, age, and population ratio. A total of 11,500 households (23 households per survey district) were surveyed using a health status questionnaire, physical examination questionnaire, and nutritional status questionnaire.

**Fig. 1:**
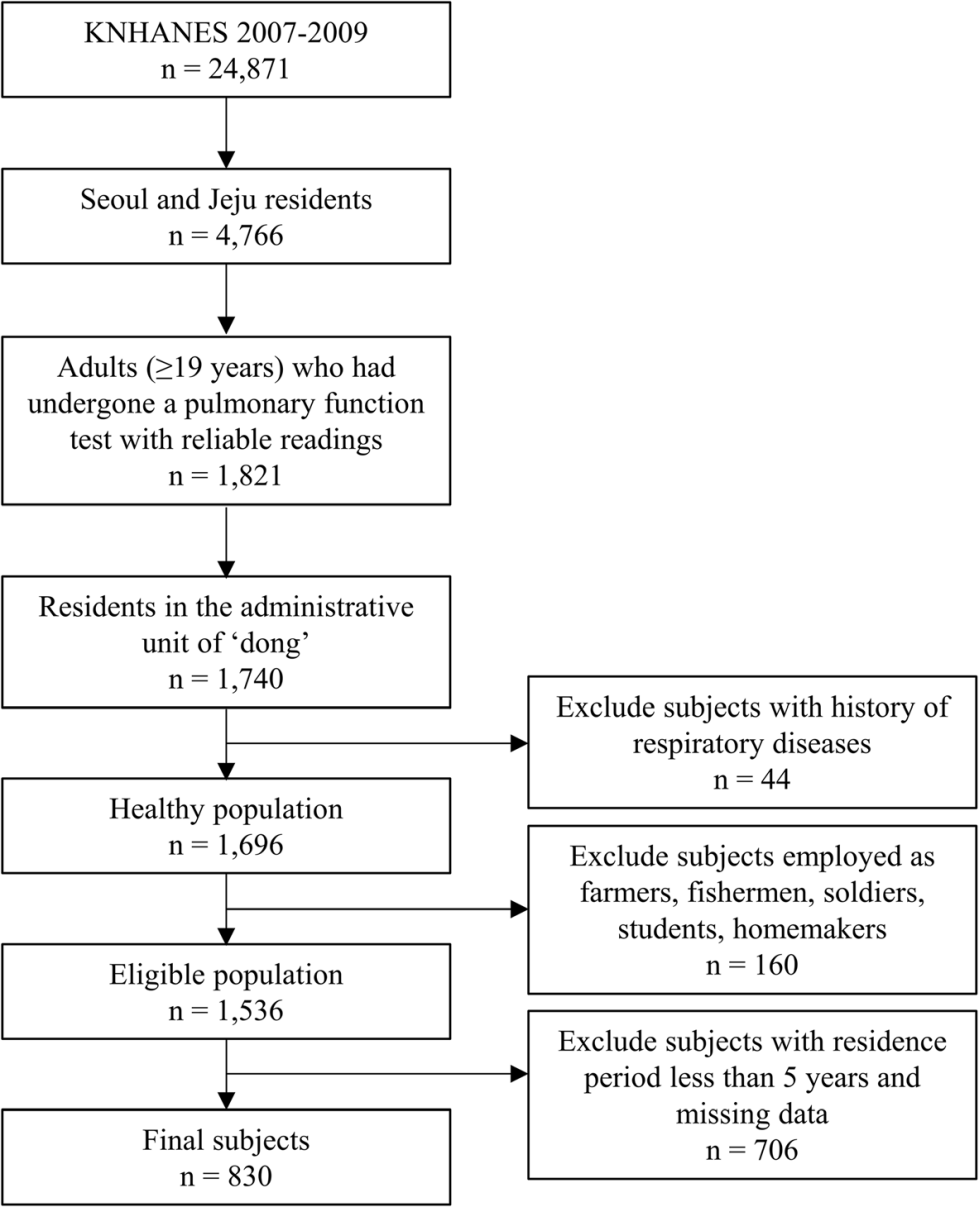
Subjects included in the present study

The present study used the data gathered from the 4th KNHANES (2007–2009), which included the health questionnaire which surveyed the period of residence in the residential area where the survey participants are living at the time the survey was conducted (Residence period). The number of respondents for each of the years in the survey was 4594 (2007), 9744 (2008), and 10,533 (2009), for a total of 24,871 respondents. Seoul which recorded the highest average annual PM_10_ concentration from 1995 to 2009, among the seven cities including six metropolitan cities (Busan, Daegu, Incheon, Gwangju, Daejeon and Ulsan) and Seoul [11]; and Jeju which recorded the lowest average annual PM_10_ concentration from 1995 to 2009, among all the cities that started to measure the PM_10_ concentration in 1995 [11], were selected for the analysis (*n* = 4766). Adults (≥19 years) who had undergone a pulmonary function test with reliable readings (*n* = 1821), and residing in the administrative unit of ‘dong,’ were enrolled in this study (*n* = 1740). Because subjects residing in the administrative units of ‘eub’ and ‘myeon,’ for which PM_10_ concentration data were not available due to lack of recordings. A further 44 subjects were excluded due to underlying respiratory diseases, including tuberculosis, asthma, bronchiectasis, lung cancer, and COPD (*n* = 1696). Subjects employed as skilled workers in the agricultural or fishing industry, actively serving in the military, or students or homemakers, were also excluded for the purpose of streamlining the occupation types into two categories (*n* = 1536). Finally, after removing the missing data and subjects with residence period less than 5 years, 830 subjects (749 Seoul residents, 340 male and 409 female; 81 Jeju residents, 39 male and 42 female) were included in the study (Fig. 1).

### Annual average PM_10_ concentration in Seoul and Jeju city

The 2009 Annual Report of Ambient Air Quality in Korea published by the National Institute for Environmental Research in 2010 was consulted to establish the difference in PM_10_ concentration between Seoul and Jeju. PM_10_ concentration data is measured by the beta ray absorption method at 27 urban air quality monitoring centers in Seoul and 2 urban air quality monitoring centers in Jeju (as of 2009) and are transmitted to the national air quality information management system via regional environmental agencies and regional public health and environment research institutes. The measurements are statistically processed at the National Institute for Environmental Research to generate a database. The average annual PM_10_ concentration data from Seoul and Jeju collected between 1995 and 2009 were used in the present study [11].

### Pulmonary function tests

Data pertaining to pulmonary function were collected from the 4th KNHANES pulmonary function test results. Pulmonary function tests were administered to individuals aged ≥19 years (excluding those with contraindications and those who refuse testing) by 4 technicians trained in test administration and test quality control. For test administration and data interpretation, the American Thoracic Society/European Respiratory Society’s 2005 standardized guidelines were followed. Pulmonary function was measured by dry rolling-seal spirometry, a type of Vmax series Sensor Medics 2130. Each subject was asked to perform a minimum of 3 acceptable maneuvers, up to a maximum of 8 maneuvers. The FEV1p and FVCp, which are the predicted values of FEV1 and FVC [12], respectively, were used as continuous variables. FEV1/FVC, which is the value obtained by dividing FEV1 by FVC, also used as continuous variable.

### Categorization of lung disease based on pulmonary function tests

All subjects were classified into one of the three mutually exclusive categories: normal, obstruction, or restriction. ‘Normal’ included subjects who had either an FEV1/FVC ≥0.70 and FVCp ≥80%. ‘Obstruction’ included subjects with FEV1/FVC < 0.70, while ‘restriction’ included subjects who had either an FEV1/FVC ≥0.70 and FVCp < 80% [13].

### Variables

Sociodemographic characteristics, health behavior data, and occupational data were collected from the 4th KNHANES’ health status questionnaire and physical measurement.

#### Socio-demographic characteristics variables

Sociodemographic characteristics included sex, age, height, bodyweight, Residence period, city of residence, educational level, and household income. Of these, age, Residence period, height, and bodyweight were analyzed as continuous variables. In stratified analysis, age was modeled as a categorical variable with levels ‘Young’, ‘Middle’ and ‘Old’ (19–38 years, 39–58 years and > 58 years, respectively). City of residence was either Seoul and Jeju. Educational level was classified as follows: ‘high’, for subjects with a high school degree or above, and ‘low’ for subjects with qualifications up to and including a middle school degree. Household income was equivalence-adjusted, and the first and second income quartiles were classified as ‘high’ while the third and fourth quartiles were classified as ‘low’.

#### Health behavior variables

Health behavior variables included smoking and drinking status. Current smokers, as well as former smokers with a history of ≥5 packs of cigarettes in their lifetime, were classified as ‘Smoker’, while lifetime never-smokers, as well as former smokers with a history of < 5 packs of cigarettes in their lifetime, were classified as ‘Non-smoker’ [14]. Drinking status was categorized as ‘heavy’ for drinking twice weekly or more and ‘social’ for drinking less than twice weekly.

#### Occupational class

To exclude any occupational effects, the survey item pertaining to the longest occupational tenure was used, and the 6th revision standard classification for occupations was consulted. Managers, professionals, office workers, and service/sales workers were categorized as ‘white collar’ workers, while technicians/equipment installers, mechanics/machine operators and assemblers, as well as unskilled workers, were categorized as ‘blue collar’ workers.

### Statistical analysis

The forth KNHANES is designed with all Koreans living in Korea as the target population and it is a complex sampling design data extracted after conducting the initial area-stratification and then the secondary stratification of households within the area. In this study, analysis was carried out considering weight, stratified variables, and cluster variables so that the sample represents the population and prevents biased outcomes. Variations in subject sociodemographic characteristics, health behavior, and occupation were analyzed using the chi-square test and a t-test. Differences in pulmonary function and categorization of lung disease according to city of residence were analyzed using the chi-square test and a t-test, applied to the subject population as a whole and to groups stratified by sex. Stratified analysis was used for analysis involving other variables potentially affecting pulmonary function which were held constant. The subjects were divided into two groups according to sex and they were stratified about age, education level, household income, occupational class, smoking status, and drinking status. Differences in pulmonary function according to city of residence were analyzed using the T-test and Mann-Whitney test. A simple comparison of Seoul and Jeju average annual PM_10_ concentration data for 1995–2009, derived from the Annual report of Air Quality, was conducted and then the repeated measures analysis of variance was used to identify differences between the two groups. All statistical analyses were performed with SPSS (version 23 for Windows, Chicago, USA) with a significance level set at α = 0.05.

## Results

### Average annual PM_10_ concentrations in Seoul and Jeju

**Fig. 2:**
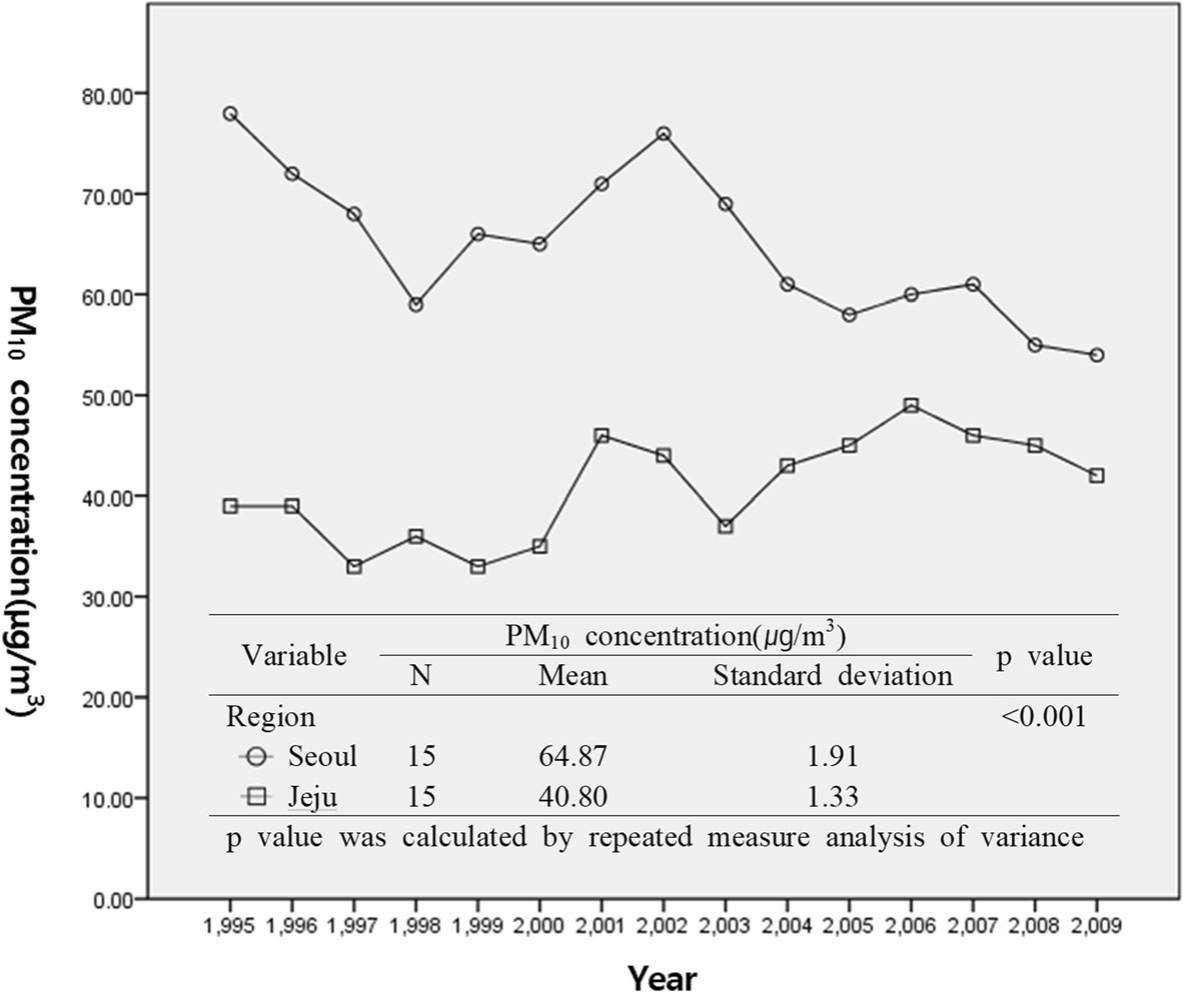
Annual mean PM_10_ concentration between Seoul and Jeju

From 1995 to 2009, the average annual concentration of PM_10_ in Seoul has always exceeded the current Korea’s air quality standard of 50 μg/m^3^ for the annual average PM_10_ concentration, but that in Jeju has never exceeded it [11]. Seoul’s 15-years average PM_10_ concentration was also higher than that of Jeju. (64.87 μg/m^3^ and 40.80 μg/m^3^, respectively). A significant difference between the two groups of Seoul and Jeju for the annual average concentration of PM_10_ was confirmed by repeated measures analysis of variance (*p* < 0.001) (Fig. 2).

### Comparison of demographic characteristics

Seoul residents had a significantly greater mean age than the Jeju residents (*p* < 0.001), while the Jeju residents had a significantly greater mean bodyweight than Seoul residents (*p* = 0.018). The educational level, household income and drinking status of residents in Seoul and Jeju were significantly different (*p* = 0.011, *p* = 0.001, *p* = 0.004, respectively). No significant differences were found between the two resident groups in terms of sex, occupational class, smoking status, height and residence period (*p* = 0.664, *p* = 0.097, *p* = 0.707, *p* = 0.093, *p* = 0.466, respectively) (Table 1).

**Table 1:** General characteristics of subjects according to city of residence

Variables	Total (*N* = 830)	Seoul (*N* = 749)	Jeju (*N* = 81)	*p* value
	N (%) or Mean (SE)	N (%) or Mean (SE)	N (%) or Mean (SE)	
Sex				0.664
Male	379 (52.4)	340 (52.2)	39 (54.1)	
Female	451 (47.6)	409 (47.8)	42 (45.9)	
Education level				0.011
Low	279 (29.1)	264 (30.1)	15 (15.0)	
High	551 (70.9)	485 (69.9)	66 (85.0)	
Household income				0.001
Low	294 (33.6)	278 (34.9)	16 (16.0)	
High	536 (66.4)	471 (65.1)	65 (84.0)	
Occupational class				0.097
Blue collar	257 (30.1)	241 (30.8)	16 (21.2)	
White collar	573 (69.9)	508 (69.2)	65 (78.8)	
Smoking status				0.707
Smoker	335 (45.4)	304 (45.6)	31 (43.2)	
Non-smoker	495 (54.6)	445 (54.4)	50 (56.8)	
Drinking status				0.004
Heavy	214 (28.5)	184 (27.8)	30 (37.8)	
Social	616 (71.5)	565 (72.2)	51 (62.2)	
Age (years)	47.3 (0.5)	47.6 (0.6)	43.7 (0.5)	< 0.001
Height (centimeter)	163.6 (0.3)	163.5 (0.4)	165.0 (0.8)	0.093
Bodyweight (kilogram)	65.7 (0.4)	65.5 (0.4)	68.4 (1.1)	0.018
Residence period (years)	12.5 (0.5)	12.6 (0.5)	11.8 (0.9)	0.466

*p* value were calculated by chi-square test or T-test to compare Seoul with Jeju

*SE* standard error

### Comparison of pulmonary function test results

Differences in FEV1p, FVCp and FEV1/FVC according to city of residence are presented in Table 2. Considering the sample as a whole, while the FVCp for Seoul residents was significantly lower than that for Jeju residents (difference: 3.48%, *p* = 0.002), the FEV1p and FEV1/FVC and did not differ significantly between the two groups of residents (*p* = 0.071, *p* = 0.167, respectively). Among male subjects, the FEV1p, FVCp and FEV1/FVC were significantly lower among Seoul residents than Jeju residents (difference: 6.99, 5.11% and 0.03, respectively; *p* < 0.001, *p* = 0.001, *p* = 0.001). Among female subjects, the FEV1p, FVCp and FEV1/FVC did not differ significantly between the Seoul residents and the Jeju residents (*p* = 0.922, *p* = 0.208, *p* = 0.971, respectively) (Table 2).

**Table 2:** The results of pulmonary function test and the number for categorization of lung disease of subjects according to city of residence

Variables	Pulmonary function tests - Mean (Standard error)			Categorization of lung disease - N (%)		
	FEV1p	FVCp	FEV1/FVC	Normal^a^	Obstruction^b^	Restriction^c^
Total						
Seoul	91.45 (0.61)	92.10 (0.56)	0.80 (0.004)	587 (81.01)	68 (7.41)	94 (11.58)
Jeju	94.81 (1.74)	95.58 (0.96)	0.82 (0.008)	69 (86.64)	5 (3.51)	7 (9.85)
*p* value	0.071	0.002	0.167	0.258		
Male						
Seoul	90.24 (0.73)	91.26 (0.68)	0.79 (0.005)	236 (76.52)	51 (10.60)	53 (12.88)
Jeju	97.23 (1.19)	96.37 (1.41)	0.82 (0.005)	34 (94.74)	4 (4.29)	1 (0.97)
*p* value	< 0.001	0.001	0.001	0.001		
Female						
Seoul	92.71 (0.82)	92.97 (0.67)	0.81 (0.004)	351 (85.63)	17 (4.12)	41 (10.25)
Jeju	92.44 (2.56)	94.81 (1.28)	0.81 (0.013)	35 (78.71)	1 (2.74)	6 (18.54)
*p* value	0.922	0.208	0.971	0.229		

*p* value were calculated by T-test to compare Seoul with Jeju, or chi-square test to compare categorization of lung disease

*FEV1p* predicted values of forced expiratory volume in 1 s, *FVCp* predicted values of forced vital capacity, *FEV1/FVC* FEV1/FVC ratio

^a^Normal included subjects who had either an FEV1/FVC ≥0.70 and FVCp ≥80%

^b^Obstruction included subjects with FEV1/FVC < 0.70

^c^Restriction included subjects who had either an FEV1/FVC ≥0.70 and FVCp < 80%

### Comparison of categorization of lung disease

Subjects’ categorization of lung disease was compared according to the city of residence via chi-square test is presented in Table 2. Considering the sample as a whole, the obstruction and restriction of Seoul residents were higher than those of Jeju residents (difference: 3.90, 1.73%, respectively), but it was not statistically significant (*p* = 0.258). Among male subjects, the obstruction and restriction of Seoul residents were higher than those of Jeju residents (difference: 6.31, 11.91%, respectively), and it was statistically significant (*p* = 0.001). Among female subjects, the obstruction of Seoul residents was only higher than that of Jeju residents (difference: 1.38%), but it was not statistically significant (*p* = 0.229) (Table 2).

### Stratified analysis of pulmonary function test results

Subjects’ pulmonary function test results were compared according to the city of residence via stratified analysis adjusting for other variables potentially affecting pulmonary function are presented in Tables 3 and 4.

**Table 3:** Stratified analysis of pulmonary function test in male subjects according to general characteristics

	N (%)		FEV1p - Mean (Standard error)			FVCp - Mean (Standard error)			FEV1/FVC - (Standard error)		
	Seoul	Jeju	Seoul	Jeju	*p*-value	Seoul	Jeju	*p*-value	Seoul	Jeju	*p*-value
Education level											
Low	89 (22.4)	3 (5.7)	88.05 (1.89)	92.71 (5.93)	0.632	89.95 (1.58)	96.40 (2.56)	0.296	0.74 (0.011)	0.75 (0.052)	0.602
High	251 (77.6)	36 (94.3)	90.81 (0.76)	97.44 (0.67)	< 0.001	91.60 (0.72)	96.37 (1.08)	< 0.001	0.81 (0.005)	0.82 (0.003)	0.021
Household income											
Low	116 (32.3)	4 (6.3)	89.33 (1.17)	95.59 (1.76)	0.474	90.93 (1.11)	83.90 (1.49)	0.357	0.78 (0.006)	0.83 (0.044)	0.861
High	224 (67.7)	35 (93.7)	90.68 (0.91)	97.38 (0.98)	< 0.001	91.42 (0.84)	97.52 (1.26)	< 0.001	0.80 (0.007)	0.82 (0.006)	0.100
Occupational class											
Blue collar	118 (33.1)	8 (22.1)	88.44 (1.49)	90.36 (1.83)	0.689	90.86 (1.22)	90.55 (2.77)	0.589	0.77 (0.010)	0.80 (0.017)	0.484
White collar	222 (66.9)	31 (77.9)	91.11 (0.79)	99.01 (1.40)	< 0.001	91.45 (0.77)	97.88 (1.44)	< 0.001	0.80 (0.006)	0.82 (0.003)	0.002
Smoking status											
Smoker	275 (80.2)	30 (78.4)	89.80 (0.89)	97.95 (1.15)	< 0.001	91.22 (0.78)	96.07 (2.44)	0.062	0.79 (0.005)	0.83 (0.006)	< 0.001
Non-smoker	65 (19.8)	9 (21.6)	91.70 (1.37)	93.60 (6.10)	0.901	91.38 (1.36)	97.91 (8.92)	0.722	0.82 (0.013)	0.76 (0.021)	0.070
Drinking status											
Heavy	141 (42.4)	19 (46.0)	90.60 (1.10)	99.43 (2.94)	0.006	92.06 (1.11)	100.88 (3.37)	0.015	0.79 (0.008)	0.81 (0.023)	0.279
Social	199 (57.6)	20 (54.0)	89.98 (0.90)	95.70 (1.75)	0.005	90.68 (0.74)	93.22 (1.66)	0.166	0.80 (0.007)	0.82 (0.007)	0.019
Age (years)											
Young (19–38)	75 (28.7)	7 (25.3)	93.35 (0.99)	99.22 (1.56)	0.993	94.24 (0.91)	98.21 (3.32)	0.523	0.84 (0.007)	0.85 (0.007)	0.993
Middle (39–58)	158 (50.4)	21 (59.3)	88.36 (1.11)	96.48 (1.77)	< 0.001	90.88 (0.92)	97.34 (1.12)	< 0.001	0.78 (0.006)	0.81 (0.007)	0.002
Old (> 58)	107 (20.9)	11 (15.4)	89.14 (1.34)	95.21 (1.12)	0.001	86.88 (1.50)	89.88 (2.81)	0.350	0.74 (0.010)	0.78 (0.037)	0.289

*p*-value were calculated by T-test or Mann-Whitney test to compare Seoul with Jeju

*FEV1p* predicted values of forced expiratory volume in 1 s, *FVCp* predicted values of forced vital capacity, *FEV1/FVC* FEV1/FVC ratio

**Table 4:** Stratified analysis of pulmonary function test in female subjects according to general characteristics

	N (%)		FEV1p - Mean (Standard error)			FVCp - Mean (Standard error)			FEV1/FVC - (Standard error)		
	Seoul	Jeju	Seoul	Jeju	*p*-value	Seoul	Jeju	*p*-value	Seoul	Jeju	*p*-value
Education level											
Low	175 (38.6)	12 (26.0)	93.28 (1.47)	87.17 (4.03)	0.159	92.20 (1.02)	84.81 (3.53)	0.047	0.79 (0.006)	0.81 (0.019)	0.270
High	234 (61.4)	30 (74.0)	92.37 (0.93)	94.44 (3.46)	0.564	93.43 (0.99)	98.60 (1.93)	0.019	0.83 (0.005)	0.81 (0.018)	0.419
Household income											
Low	162 (37.8)	12 (27.5)	91.15 (1.53)	85.24 (2.81)	0.068	90.87 (1.17)	83.97 (2.17)	0.006	0.80 (0.006)	0.83 (0.021)	0.172
High	247 (62.2)	30 (72.5)	93.64 (0.97)	95.91 (4.11)	0.593	94.24 (0.91)	100.02 (2.06)	0.012	0.82 (0.006)	0.80 (0.017)	0.346
Occupational class											
Blue collar	123 (28.2)	8 (20.1)	91.60 (1.65)	85.70 (3.89)	0.448	91.86 (1.43)	84.44 (2.54)	0.308	0.80 (0.006)	0.80 (0.017)	0.773
White collar	286 (71.8)	34 (79.9)	93.14 (0.95)	94.20 (3.56)	0.774	93.41 (0.81)	97.51 (1.84)	0.044	0.82 (0.005)	0.82 (0.014)	0.740
Smoking status											
Smoker	29 (7.8)	1 (1.7)	90.31 (2.39)	97.45 (0)	0.800	90.98 (2.18)	114.60 (0)	0.133	0.83 (0.010)	0.73 (0)	0.133
Non-smoker	380 (92.2)	41 (98.3)	92.97 (0.88)	92.33 (2.72)	0.822	93.19 (0.77)	94.35 (1.59)	0.515	0.81 (0.005)	0.81 (0.012)	0.806
Drinking status											
Heavy	43 (11.8)	11 (28.2)	91.38 (1.93)	93.34 (0.99)	0.368	92.85 (1.55)	98.28 (0.84)	0.003	0.83 (0.018)	0.80 (0.011)	0.231
Social	366 (88.2)	31 (71.8)	92.88 (0.87)	92.02 (3.49)	0.812	92.99 (0.70)	93.17 (2.27)	0.940	0.81 (0.004)	0.82 (0.015)	0.654
Age (years)											
Young (19–38)	64 (19.4)	13 (35.0)	91.77 (1.28)	93.61 (2.54)	0.521	93.12 (1.30)	98.40 (3.01)	0.111	0.85 (0.008)	0.82 (0.020)	0.127
Middle (39–58)	232 (58.8)	22 (48.2)	92.06 (1.01)	91.10 (5.24)	0.858	93.72 (1.04)	92.44 (4.85)	0.797	0.81 (0.005)	0.81 (0.012)	0.728
Old (> 58)	113 (21.7)	7 (16.8)	95.32 (1.86)	91.95 (5.65)	0.436	91.13 (1.26)	86.36 (7.57)	0.430	0.78 (0.008)	0.78 (0.009)	0.906

*p*-value were calculated by T-test or Mann-Whitney test to compare Seoul with Jeju

*FEV1p* predicted values of forced expiratory volume in 1 s, *FVCp* predicted values of forced vital capacity, *FEV1/FVC* FEV1/FVC ratio

In male subjects only, the FEV1p, FVCp and FEV1/FVC of Seoul residents with education level is ‘high’ (*p* < 0.001, *p* < 0.001, *p* = 0.021, respectively), occupational class is ‘white collar’ (*p* < 0.001, *p* < 0.001, *p* = 0.002, respectively) or age is ‘middle’ (*p* < 0.001, *p* < 0.001, *p* = 0.002, respectively) were significantly lower than those of Jeju residents. The FEV1p and FVCp of Seoul residents with household income is ‘high’ (*p* < 0.001, *p* < 0.001, respectively) or drinking status is ‘heavy’ (*p* = 0.006, *p* = 0.015, respectively) were significantly lower than those of Jeju residents. The FEV1p and FEV1/FVC of Seoul residents with smoking status is ‘smoker’ (*p* < 0.001, *p* < 0.001, respectively) or drinking status is ‘social’ (*p* = 0.005, *p* = 0.019, respectively) were significantly lower than those of Jeju residents. The FEV1p of Seoul residents with age is ‘Old’ was significantly lower than that of Jeju residents (*p* = 0.001) (Table 3).

In female subjects only, the FVCp of Seoul residents with education level is ‘high’, household income is ‘high’, occupational class is ‘white collar’ or drinking status is ‘heavy’ was significantly lower than that of Jeju residents (*p* = 0.019, *p* = 0.012, *p* = 0.044, *p* = 0.003, respectively), but the FVCp of Seoul residents with education level is ‘low’ or household income is ‘low’ was significantly higher than that of Jeju residents (*p* = 0.047, *p* = 0.006, respectively) (Table 4).

## Discussion

The present study, which was based on the 2009 Annual Report of Ambient Air Quality in Korea and the 4th KNHAENS data, found a significant difference in pulmonary function test results between Seoul and Jeju residents with different average annual concentration of PM_10_ (Table 2). After adjusting for variables potentially affecting the pulmonary function test results through stratified analysis, in male subjects, pulmonary function results of Seoul residents were significantly lower than those of Jeju residents (Table 3), but in female subjects, the FVCp of Seoul and Jeju residents varied depending on the stratifying variables (Table 4).

Airborne particulate matter, including PM_10_ which has settled and accumulated in the lung via the mechanisms of impaction, sedimentation, diffusion [15], is eliminated by the body’s defense mechanisms, namely, lung epithelial fluid and alveolar macrophages [16–18]. However, as air pollution intensifies, the phagocytic and microbicidal functions of alveolar macrophages diminish [19] and the radical oxygen and proteinase resulting from the activation of alveolar macrophages causes inflammation in the lung [18, 20]. The reduced pulmonary function of the Seoul residents relative to the Jeju residents may be attributed to this mechanism of lung inflammation and damage occurring with prolonged exposure to a high level of PM_10_ concentration.

Lower socioeconomic status is associated with an increased risk for developing COPD [21]. A longitudinal Study in firefighters have shown that occupational exposures reduce pulmonary function [22], and an analysis of the large U.S. population-based National Health and Nutrition Examination Survey III estimated the fraction of COPD attributable to workplace exposures was 19.2% overall, and 31.1% among never-smokers [23]. In the stratified analysis of the present study, among male subjects, the pulmonary function test results of Seoul residents with education level is ‘high’, household income is ‘high’ or occupational class is ‘white collar’ were significantly lower than those of Jeju residents (Table 3). These results were in good agreement with the purpose of this study because they showed a more significant correlation in the less affected group of other disturbance variables that may affect the pulmonary function test results.

Cigarette smokers have a higher prevalence of respiratory symptoms and a greater annual rate of decline in FEV1 [24]. Those who stop smoking will experience only a small recovery in pulmonary function level, but they will cease to lose pulmonary function at an accelerated rate [25]. In the stratified analysis of the present study, among male subjects, the pulmonary function test results of Seoul residents with smoking status is ‘Smoker’ were significantly lower than those of Jeju residents (Table 3). These results suggest that smoking may be a confounding factor for differences in pulmonary function between Seoul and Jeju residents. However, this result may also indicate that smokers are more sensitive to PM_10_ exposure. Lindgren et al. [26] examined associations between residential traffic and asthma and COPD in adults in southern Sweden. In a stratified analysis for smoking, the authors found that the effects of traffic exposure were more pronounced for smokers than for non-smokers, for both COPD diagnosis and bronchitis symptoms. XU et al. [27] investigated the hypothesized synergistic effects of air pollution and personal smoking on pulmonary function in a random sample of 3287 adults (40–69 years of age) who resided in residential, industrial, and suburban areas in Beijing. The authors found that long-term exposure to high levels of particulate in Beijing was associated with significantly reduced pulmonary function in both never smokers and smokers, and the associations were significantly greater among smokers than among never smokers, indicating a synergistic effect of air pollution and personal smoking on adult pulmonary function.

The effects of drinking on pulmonary function are still controversial. An alcohol consumption of > 350 g a week significantly accelerated the loss of FEV1 and the loss of FVC with 5 years observation time controlling for smoking [28]. In a 10 years study [29], cross sectional studies showed that increased alcohol consumption was significantly associated with impaired age adjusted and height adjusted FEV1 in 328 policemen, but in the longitudinal analyses, there was no relation between alcohol consumption and FEV1 decline. Twisk et al. [30] found a positive relation with alcohol consumption and FVC and FEV1 in a young population (ages 13–27 years). In the stratification analysis of the present study, among male subjects, the pulmonary function test results of Seoul residents regardless drinking status were significantly lower than those of Jeju residents (Table 3), it is not clear that drinking will affect lung function deterioration due to PM_10_ exposure.

It is known that pulmonary function is increased to 27 years for male and 20 years for female and decreases with increasing age [31]. In the present study, predicted values of pulmonary function were used to adjust for age affecting pulmonary function, but stratified analysis for age was performed because the most widely recognized risk factors for COPD are increasing age [32]. Among male subjects, the difference in the FEV1p between Seoul and Jeju residents was more prominent in ‘Middle’ and ‘Old’ age groups, and the FVCp and FEV1/FVC between Seoul and Jeju residents was more prominent in ‘Middle’ age groups (Table 3). These results were in good agreement with the purpose of this study because they showed a more significant correlation in the older age groups that likely to have been exposed to PM_10_ for longer periods than younger age group. Aging is associated with accumulation of particles and metals in the mammalian lung [33–35], and exogenous carbonaceous particles appear to accumulate progressively with age, but accurate quantification has not been achieved [36]. The effects of air pollution material on age-associated changes have been studied in rats. Chen et al. [37] experimented with young, adult, and old rats physiologically inhaled air containing aerosol of manufactured SiO_2_ nanoparticles (24.1 mg/m^3^; 40 min/day) for 4 weeks. Inhalation of SiO_2_ nanoparticles under identical conditions caused pulmonary alterations in old rats, yet less change in young and adult rats, including pulmonary inflammation. But Increased susceptibility to PM_10_ exposure results from aging is not clear in human, so it may be necessary to further investigate the vulnerability of PM_10_ according to age.

In the present study, there were no significant differences in pulmonary function in female between the Seoul residents and the Jeju residents (Table 2), and in stratified analysis, the FVCp of Seoul and Jeju residents varied depending on the stratifying variables (Table 4). These results are thought to have occurred for the following reasons. First, the result may be attributed to the difference between the sexes in sensitivity to PM_10_. Kim et al. [38] studied 22 men and women (11 male and 11 female subjects) to examine the difference between the sexes in location within the lungs where inhaled airborne particulate matter settles. The results showed that, airborne particulate matter with aerodynamic diameter of 3 and 5 μm tended to be accumulated shallow region in female’s lungs compared with male. A 3-year cohort study [39] by the Ministry of Environment analyzed pulmonary function among residents of Seoul and its neighboring areas where air pollution is high. The results showed an annual decrease in FEV1 by 78 mL in men and 28 mL in women, clearly indicating a lower rate of decline in pulmonary function per annum among women. Second, it is possible that age and socioeconomic status served as a confounding variable. Lower socioeconomic status and age may be the cause of decreased pulmonary function [21, 31, 32]. In female group, the effect was greater in Jeju than in Seoul. As a result, female with low educational level and household income had higher pulmonary function in Seoul than Jeju, which was in contrast to the results of higher socioeconomic status group (Table 4). Finally, the difference in annual mean PM_10_ concentrations between Seoul and Jeju may not be large enough to change the lung function of non-smoking female. Smokers may have more severe pulmonary function reductions by exposure to PM_10_ than non-smokers [26, 27]. However, in the present study, the proportion of smokers in female is much lower than that of non-smokers (Table 4). Thus, for female group with a lower percentage of smokers than male group, there may not have been a significant change in pulmonary function over the long term exposure of PM_10_.

Although the present study was based on survey data collected from a nationally representative sample, interpretation of these findings should take into account the following limitations. First, the measurement and exposure assessment of PM_10_ concentration may not have been performed properly. It was not feasible to assess individual exposure of Seoul and Jeju residents to PM_10_, therefore, the Annual Report of Ambient Air Quality in Korea published by the Ministry of Environment was used. Unfortunately, it is not clear whether the number and location of the measuring centers across Seoul and Jeju were sufficient to collect data representative of the entire cities. Second, Although the KNHANES’ health status questionnaire on residence period was used to assess the exposure of PM_10_ to subjects, it did not provide accurate information on how long the subject actually lived in Seoul or Jeju. Because the 4th KNHANES only provides information about how long the subjects lived in the house in question at the time of the survey, so the residence period in that area can be underestimated. For this reason, the number of final subjects was reduced when subjects were limited to those with residence periods of 5 years or more. When the number of subjects in the stratified analysis was too small to satisfy the normality, a nonparametric statistical method was used. In this case, it was difficult to obtain statistically significant results. As a representative example, mean values of FEV1 in Seoul and Jeju were different in the young age group of male subjects, but no statistically significant results were obtained (Table 3). Unfortunately, the KNHANES’ health status questionnaire also does not contain items pertaining to past residence. Therefore, the exposure-reaction association is also unclear. Third, to exclude any occupational effects, occupations were classified into ‘white collar’ and ‘blue collar’ based on the longest occupational tenure classification. However, because the data were collected via a questionnaire, individual exposure to PM_10_ at work could not be assessed adequately. Fourth, stratified analysis was conducted to exclude the effect on smoking, but other factors such as age were not adjusted together. In general, increasing age are known to cause a decrease in FEV1 [31, 32]. The average age of male smokers living in Jeju was lower than that of nonsmokers (42.44 years, 49.01 years, respectively). For this reason, smokers living in Jeju may have abnormally higher FEV1p than non-smokers (Table 3). Therefore, age may be a confounding variable, and it may not be possible to precisely exclude the effects of smoking on lung function. As a result, significant differences in pulmonary function may have occurred only in male smokers in Seoul and Jeju (Table 3). Furthermore, other air pollutants, including ozone and nitrogen dioxide, known to contribute to reduced pulmonary function [40, 41] were not evaluated or adjusted for in the present study. Eventually, it is important to note that due to the cross-sectional design of this study, unlike longitudinal studies, it does not establish a clear causal association between the variables.

Despite these limitations, the main contribution of the present study is that it is one of the few Korean studies comparing pulmonary function between residents of two cities with vastly different PM_10_ measurements. The finding that individuals residing in areas characterized by high levels of PM_10_ may have significantly diminished pulmonary function is supported by the fact that the analysis adjusted for potentially confounding socioeconomic variables (occupational class, household income and educational level), Health behavior variables (smoking and drinking status) and biological variable (age and sex).

## Conclusion

These results indicate that the pulmonary function of Seoul residents was significantly lower than that of Jeju residents, where the average annual PM_10_ concentration is considerably lower. Therefore, national and local authorities should continue to implement strategies to reduce PM_10_ in the air, which have a deleterious effect on pulmonary health. It is important to conduct a prospective cohort study in order to determine the association between PM_10_ and reduced pulmonary function and other health hazards.

## Abbreviations

%: Estimated percentage
CI: Confidence interval
COPD: Chronic obstructive pulmonary disease
FEV1/FVC: FEV1/FVC ratio
FEV1p: Predicted values of forced expiratory volume in 1 s
FVCp: Predicted values of forced vital capacity
Jeju: Jeju special self-governing province including Jeju city and Seogwipo city
KCDC: Korea Centers for Disease Control and Prevention
KNHANES: Korea National Health and Nutrition Examination Survey
PM10: Airborne particulate matter with an aerodynamic diameter less than or equal to 10 μm
Residence period: The duration the period of residence in the residential area where the survey participants are living at the time the survey was conducted
SALLIA: In the German study on the Influence of Air Pollution on Lung Function, Inflammation, and Aging
SAPALDIA: The Swiss Study on Air Pollution and Lung Disease in Adults
SE: Standard error
Seoul: Seoul special city
